# Abortion and Lethal Septicaemia in Sows Caused by a Non-ST194 *Streptococcus equi* subsp. *zooepidemicus*

**DOI:** 10.1155/2024/4008946

**Published:** 2024-07-23

**Authors:** Ervin Albert, István Emil Kis, Krisztián Kiss, Katalin K-Jánosi, Matheus de Oliveira Costa, György Tolnai, Imre Biksi

**Affiliations:** ^1^ Department of Pathology University of Veterinary Medicine Budapest, Üllő, Hungary; ^2^ Institute of Metagenomics University of Debrecen, Debrecen, Hungary; ^3^ SCG Diagnosztika Kft., Délegyháza, Hungary; ^4^ Department of Large Animal Clinical Sciences Western College of Veterinary Medicine University of Saskatchewan, Saskatoon, Canada; ^5^ Department of Population Health Faculty of Veterinary Medicine Utrecht University, Utrecht, Netherlands; ^6^ Animal Health Centre of Szabadhegy, Győr, Hungary

## Abstract

Outbreaks of zoonotic *Streptococcus equi* subsp. *zooepidemicus* (SEZ) have caused severe epidemics in the pig sector since the 1970s in Southeastern Asia, China, and more recently North America. Cases of high mortality caused by peracute septicaemia were all attributed to strains of a highly virulent clonal lineage belonging to the sequence type (ST) 194. In Europe, only two outbreaks have been reported with similar features, caused by other sequence types. In August 2023, a febrile disease followed by abortion and subsequent death was observed among sows kept in a small-scale organic pig farm in West Hungary. Symptoms, pathological lesions, and microbiological findings were suggestive of septicaemia from bacterial origin caused by SEZ. According to the results of the routine laboratory testing, no other relevant infectious agents were involved. Whole-genome sequence analysis assigned the examined strains to ST138, unrelated to any of the European isolates. It also revealed a few common SEZ virulence genes, compared to the highly virulent ST194 strains. A sudden weather change and subsequent extremely high average daily temperature before the outbreak could be identified as the only predisposing factor. The immediate antibiotic treatment and applied biosecurity measures might have helped to restrict and terminate the outbreak. To our knowledge, this is the first report on abortion and lethal septicaemia in sows from Central and Eastern Europe. The results call attention to the potential of non-ST194 SEZ strains to cause outbreaks in pig farms.

## 1. Introduction

*Streptococcus equi* subsp. *zooepidemicus* (SEZ) is a Gram-positive, beta-haemolytic, Lancefield group C *Streptococcus* and a common colonizer of most warm-blooded species, including humans [1]. The bacterium is considered an opportunistic pathogen with a wide host range, primarily affecting horses, where associated disease may include rhinitis, pleuropneumonia, placentitis, foal septicaemia, and arthritis [2, 3]. SEZ also causes respiratory disease in dogs [4] and cats [5] as well as septic infection in guinea pigs and llamas [6, 7] and has been reported as an emerging zoonotic pathogen with various clinical manifestations [8, 9].

SEZ is suggested to be part of the resident flora on mucous membranes of pigs [1]; however, certain strains may result in epizootic disease with high mortality rates. In the 1970s, more than 300,000 pigs were lost due to an outbreak caused by a virulent strain in Sichuan, China [10], and the pathogen has remained a concern in the Southeast Asian region since then [11]. During 2019, SEZ outbreaks were also recorded in Canada and the USA, resulting in significant animal and economical losses in both countries [12, 13].

In the case of high mortality outbreaks, the animals showed no clinical signs few hours previous to their sudden death [12, 13]. Affected sows became weak and lethargic, and they showed dyspnoea as well as ataxia could be observed if the animals would be able to move, while some of them developed the same clinical signs after aborting [12, 13, 14]. Lethargy, elevated respiratory rate with dyspnoea, and marked abdominal breathing as well as fever were observed during experimental infections of swine with SEZ [15, 16].

During natural cases of SEZ infection, the gross pathology of dead sows revealed typical findings of sepsis [12, 17]. The most consistent gross pathological findings were enlarged, red-to-tan lymph nodes as well as haemorrhagic lymphadenopathy [17] and splenomegaly [18], while in some cases, fibrinous exudate on the epicardium and splenic capsule [13], rhinitis, pulmonary and gall bladder oedema [17], polyarthritis, bronchopneumonia, pleuritis, endocarditis, and meningitis, as well as polyserositis, were described [11, 19, 20].

No severe gross lesions could be observed in the first 24 hr post-infection, in the case of experimental infection of sows with SEZ. Mild-to-moderate haemorrhage or hyperaemia as well as moderate oedema and reactivity could be observed of the submandibular lymph node, mild focal necrosis of the spleen, and small consolidated areas in the lungs, and serous exudate in the abdominal cavity and pericardium was found [16, 21]. Microbiological laboratory examination resulted viable SEZ at all time points, from tonsils samples of all experimentally infected pigs [21].

During investigation of bacterial disease outbreaks, the tracing of evolutionary origin and spread of pathogens frequently involves genomic approaches, like the widely used method of multilocus sequence typing (MLST). In SEZ, combinations of the allelic variants of seven, predefined housekeeping gene loci determine multilocus sequence types (ST). Assignment of isolates to the same ST by the dedicated MLST database can support their close evolutionary relatedness or may be indicative of their host or infection site preferences [22]. Isolates from North American and Chinese high mortality cases belonged to the ST194, and they also formed a distinct subcluster among other SEZ isolates when investigated by whole-genome based phylogeny [12, 18]. Challenge trials later confirmed the extreme virulence potential of this sequence type in pigs [16], while genome analyses suggested the presence of an M-like protein variant, and a combination of genomic islands unique to this lineage [18, 23].

Although the newly discovered virulence factors of ST194 may contribute to the increased pathogenicity of the isolates [13, 24, 25], SEZ belonging to other lineages can also cause severe disease in pigs, demonstrated by sporadic cases and isolated outbreaks [14, 18, 20]. Interestingly, SEZ has also been reported in Europe as an emerging pathogen in pigs; however, none of the outbreak isolates could be associated with ST194. High mortality of gestating sows was recorded in two loose housed pig herds in the Netherlands in 2019, and the isolated SEZ was proved to be a new, non-ST194 sequence type [26]. Another study from 2023 described high morbidity and mortality among sows due to septicaemia in a German conventional farm and confirmed the causative agent as a new sequence type of SEZ, ST524 [20].

Here, we report abortions and increased mortality of sows due to SEZ septicaemia in a West Hungarian pig farm and the first known case in the Central and Eastern region of Europe.

## 2. Materials and Methods

### 2.1. Farm Description and Data Collection

In August 2023, a febrile disease, followed by abortion and subsequent death, was observed among sows kept in an organic pig farm in West Hungary. In accordance with its organic certification, the farm applied a combination of free range and closed housing, where animals from all stables had free access to uncovered, open-air paddocks and straw bedding. The unit operated “farrow to finish”; i.e., all ages and production groups were reared on the same farm. The breeding herd consisted of sows (*n* = 184), boars (*n* = 4), gilts (*n* = 15), and 83 recently purchased replacement gilts in quarantine. Gestating sows were housed in one stable, divided into 12 groups of maximum 16 animals per paddock. Farrowed sows were kept individually together with their offsprings for 4 weeks in two separated farrowing units, each accommodating a maximum of 32 animals at a time. Next, sows and offsprings were relocated to the nursery, where they were kept in groups of four to five sows per paddock until weaning in the 7th week of piglets' life. After weaning, feeders were moved to a separate fattening unit and left the farm at the weight of ~50 kg for finishing in Austria. The farm maintained a continuous flowthrough of animals in the different compartments. The annual sow mortality and abortion rate of the farm was 5–6 (~3%) and 4–5 (~2.5%) sows per year, respectively.

All production groups but sucking piglets were vaccinated against *Erysipelothrix rhusiopathiae* (ER): sows in every lactation, 1 week before weaning, gilts 6 weeks and 2 weeks before the first mating, boars in every 6th month and fatteners in the 8–10th weeks of life. Porcine parvovirus (PPV1) vaccine was administered to sows and gilts in combination with the ER vaccination. Piglets were vaccinated against *Mycoplasma hyopneumoniae*, porcine circovirus (PCV) 2, and *Lawsonia intracellularis* within the first 4 weeks of life. A vaccine against F4^+^ and F18^+^*Escherichia coli* was administered to piglets 5–7 days before weaning.

Cleaning and disinfectant products were use as listed in the Commission Implementing Regulation (EU) 2021/1165 (https://eur-lex.europa.eu/eli/reg_impl/2021/1165/oj, last accessed 25 February 2024). Feed could be purchased from certified organic origin, and medication, apart from vaccination, was restricted according to the Regulation (EU) 2018/848 of the European Parliament and of the Council (https://eur-lex.europa.eu/eli/reg/2018/848/oj; last accessed 25 February 2024). Animals received water both from a farm-owned well and the local public drinking water network.

In search of possible predisposing factors, data were collected on sudden changes, introduction of new animals, and the history of intermittent diseases. Changes in diet, water supply or other relevant housing technology parameters, staffing, and extreme weather conditions were all considered as possible stress factors. Weather data were requested from the meteorologic data base of the Hungarian Meteorological Service (https://odp.met.hu/climate/observations_hungary, last accessed 22 February 2024).

### 2.2. Laboratory Investigations

Serum samples from affected sows (*n*_native_ = 5, *n*_EDTA_ = 4) were ELISA tested for antibodies against suid herpesvirus 1 (SuHV-1, Aujeszky's disease virus), *Brucella suis*, porcine reproductive and respiratory syndrome virus (PRRSV), as well as tested with microscopic agglutination test (MAT) for antibodies against *Leptospira* species, and PCR tested for PRRSV, African swine fever virus and porcine teschovirus (contagious porcine paralysis). These examinations were carried out in the Veterinary Diagnostic Directorate of the National Food Chain Safety Office (Budapest, Hungary).

Organ collection of a selected sow (abdominal and thoracis viscera) and foetuses (*n* = 2) of another sow was sent for laboratory testing to the Production Animal Diagnostic Centre (University of Veterinary Medicine Budapest, Üllő, Hungary). A pool of sow and foetal tissues was tested for PRRSV, PCV-2, PCV-3, *Leptospira* sp., SuHV-1, PPV1, *Glässerella parasuis*, and *Brucella* sp. by real-time PCR.

Routine aerobic bacterial examination of the sow organ collection and foetuses was carried out on Columbia 5% sheep blood agar (Biolab, Budapest, Hungary) at 37°C for 24 hr, under normal atmospheric conditions. The isolates were identified on a genus level with basic biochemical tests and with Gram-staining. The species- and subspecies-level identification were carried out using API 20 Strep biochemical test kit (bioMérieux, Belgium) and confirmed by a standard PCR that differentiates between the two subspecies, *S. equi* subsp. *zooepidemicus* and subsp. *equi* [27].

Further, real-time PCR was applied to detect the *szM* gene variant, considered typical for the highly virulent SEZ strains of ST194, previously recovered from pigs [28]. Shortly, the isolation of bacterial genomic DNA was performed from pure cultures using a direct lysis method (PrepMan Ultra, Thermo Fisher Scientific, Warrington, United Kingdom), according to the manufacturer's instruction. The real-time PCR reaction mixture consisted of 0.5 mM of each of the forward and reverse primers, 0.25 mM of the dually labelled fluorescent probe, and 2 *μ*L template DNA in a total volume of 12 *μ*L. The reactions were performed on a LightCycler 480 II instrument (Roche, Rotkreuz, Switzerland), applying the following cycling conditions: 95°C for 2 min followed by 40 cycles of 95°C for 15 s and 60°C for 30 s.

Two representative isolates (S23-21852 and S23-21854) were selected for antibiotic susceptibility testing and whole-genome sequencing (Table 1). Susceptibility testing was performed for 19 antimicrobial agents by using a commercial microdilution assay (AST-MIC; Merlin Diagnostika GmbH, Berlin, Germany), and breakpoints were evaluated automatically by the manufacturer's software using CLSI standards [29]. For antibiotics not listed in the above mentioned CLSI standards, the SIR assessment was carried out in accordance with the recommendations of the Working Group on Antibiotic Resistance, German Veterinary Medical Society (DVG). The full list of tested antimicrobial compounds and the results can be found in Table 2.

### 2.3. Whole-Genome Sequencing (WGS) and Bioinformatic Analysis

The two selected SEZ isolates were whole genome sequenced in the sequencing facility of the Biomi Kft. (Gödöllő, Hungary) on a MiSeq platform with MiSeq Reagent Kit V2 using paired-end 250 bp chemistry, as previously described [30]. Draft genomes were assembled and annotated, and phylogeny was inferred using the bioinformatic package of the Bacterial and Viral Bioinformatics Resource Centre (BV-BRC) [31], including publicly accessible genomes (*n* = 34), further two from a previous Canadian study [12], and an isolate from a recent Dutch pig outbreak [26] in the analysis. A phylogenetic tree was reconstructed using the alignment of 1,000 randomly picked, shared single-copy amino acid and nucleotide sequences of the selected genomes by the Bacterial Genome Tree Service in BV-BRC, and was subsequently visualized and annotated in iTOL [32]. Whole-genome multilocus sequence typing and the search for 14 previously described putative virulence determinants (Table S1) [33, 34, 35] by nucleotide BLAST were also performed in BV-BRC. The average nucleotide identity (ANI) of the selected genome pairs was calculated by using the OrthoANI Calculator webtool [36]. Possible resistance determinants were investigated by using the ResFinder 4.5 webtool [37].

## 3. Results

### 3.1. Case Presentation and Clinical Findings

During the 2 weeks of the outbreak, no other production group was affected, only the sows. Fever (>40°C), weakness and subsequent hindlimb paralysis, and abortion as well as dyspnoea and complete recumbency could be observed in the affected sows prior to death. During the outbreak, altogether eight (4.3%) sows died of which five (2.7%) pregnant animals aborted prior to their death. These numbers exceeded the usual five to six losses per year and were equal to the average annual abortion rate, respectively.

Diseased animals were isolated immediately. For treatment, amoxicillin trihydrate was applied *per os* (Amoxid 800 mg/g AUV powder, Dunavet-B, Dunaföldvár, Hungary), in the dosage of 20 mg/kg bodyweight for 5 consecutive days, individually calculated for all sows. The entire breeding flock was treated. Non-feeding animals were given the same active substance (Veyxyl LA 200 inj., Veyx-Pharma, Schwarzenborn, Germany) parenterally also for 5 days. Intensified hygienic control of workers and visitors were applied by using ethanol-based hand sanitizers and germicidal soaps and installing disinfection mats in every passageway. To disinfect vehicles, wheel washers and disinfection gates were operated with preparations containing quaternary ammonium compounds and glutaraldehyde (Perfect Kombicid, Alpha-Vet Állatgyógyászati Kft., Székesfehérvár, Hungary). The same preparation was used for disinfecting the surfaces of stables and other premises after cleaning with a high-pressure washer. Restriction and control of traffic were also implemented on the farm immediately after the disease outbreak. Strict hygienic control measures were maintained until the negative results of subsequent microbiological examinations.

### 3.2. Gross Pathological Observations

Five out of the eight dead sows were necropsied on the farm and revealed late-gestation foetuses in the uterus of three gestating animals, cranioventral lung consolidation in three animals, and in one case, severe pleuritis and peritonitis. The organs of this sow were sent to the Production Animal Diagnostic Centre, where their macroscopic examination further revealed splenomegaly and croupous bronchopneumonia. In the two submitted aborted foetuses from an unrelated sow, only sings of bronchopneumonia could be noted.

### 3.3. Laboratory Findings

The serologic testing for SuHV-1 infection, brucellosis, PRRS, leptospirosis, and PCR testing for PRRSV, African swine fever virus and porcine teschovirus (contagious porcine paralysis), yielded negative result in all five cases. Also, the PCR-based testing of the pooled foetal tissue sample was negative for PRRSV, PCV-2, PCV-3, *Leptospira* sp., SuHV-1, PPV1, *Glässerella parasuis*, and *Brucella* sp.

The routine aerobic bacterial culture from sow spleen (*n* = 1) and liver (*n* = 1) samples and lungs from both foetuses (*n* = 2) resulted pure culture of beta-haemolytic, small, greyish white, mucous colonies. Based on routine biochemical tests and Gram-staining, the isolated colonies were identified as *Streptococcus* sp. Further biochemical speciation using the API 20 Strep biochemical test kit identified the isolates as *S. equi* subsp. *zooepidemicus* (SEZ), which was corroborated by PCR. Results are summarized in Table 1.

The strains were found to be susceptible to most antimicrobial compounds tested, including beta-lactam antibiotics. One of the isolates was apparently resistant to enrofloxacin ( = 2 mg/L), while intermediate resistance was reported in the other ( = 0.5 mg/L). Further on, intermediate resistance was reported in the case of tetracycline, neomycin in both isolates (Table 2), though no known resistance determinants could be identified in the genomes.

### 3.4. Whole-Genome Sequencing and Bioinformatic Analysis

**Figure 1:**
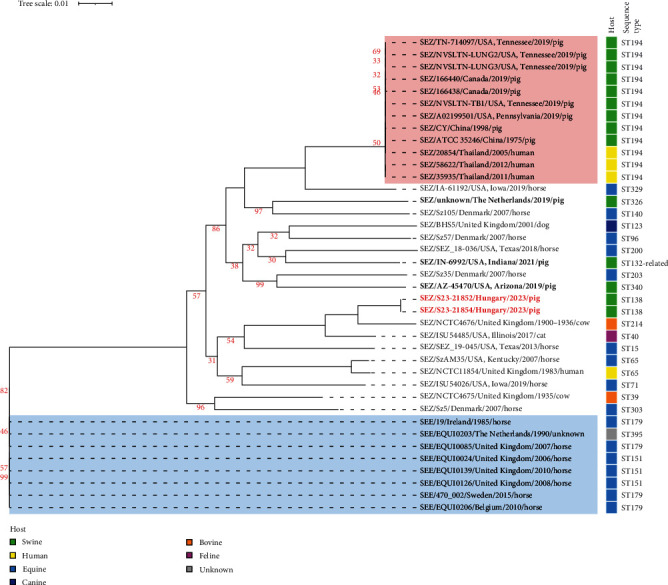
Phylogenetic comparison of *S. equi* subsp. *zooepidemicus* (SEZ) whole-genome sequences obtained from the outbreak described in this study (red, bold characters) and other *S. equi* isolates from public databases. The tree was constructed based on codon differences of 1,000 randomly chosen shared genes of the strains within the BV-BRC online platform [31]. Tree scale bar indicates the number of codon substitutions per site. Only bootstrap values <100 are shown. Coloured shadings indicate the highly clonal subpopulation of virulent *S. equi* subsp. *zooepidemicus* sequence type 194 (red) and the distantly related cluster of *S. equi* subsp. *equi* (SEE) sequences (blue). Non-ST194 *S. equi* subsp. *zooepidemicus* strains from pigs are bolded.

The WGS of the S23-21852 and S23-21854 SEZ isolates resulted 572,857 and 786,119 reads, respectively, both yielding a minimum average coverage of 120x. Their assembled genome lengthS were 2,103,181 and 2,103,657 bp, respectively, and both genomes had an average GC content of 41.54%. They showed 100% pair-wise whole-genome average nucleotide identity (ANI) score when compared to each other and were both assigned to MLST sequence type 138, confirming their clonality. Codon-based phylogenetic analysis clearly sets these two strains apart from other pig isolates (Figure 1), including the highly clonal subpopulation of ST194 strains causing epizootic outbreaks in Southeast Asia and North America. This notion was reinforced by the ANI scores: strains isolated in this study were 97.01% homologous to the type strain ATCC 35246 (ST194). Raw reads of the sequenced isolates are available in the NCBI Sequence Read Archive database under the project number PRJNA1086057.

Of the 14 putative virulence genes which were previously suggested to be involved in the hypervirulent nature of ST194 strains, some were missing from our isolates, or a different variant was presented (Table S2). The BLAST search identified two M-like protein genes, *szP* and an *szM* variant, another surface-anchored protein gene, encoding the streptococcal protective antigen Z (*spaZ*) and a type D streptodornase gene (*sdzD*). Of these, all but the ATCC 35246-type *szM* variant seemed to be ubiquitous in SEZ isolates. The *szM* gene sequence in our isolates had 86.1% pairwise identity compared to that of the ST194 type strain ATCC 35246 and consequently considered as a different one. This was in line with the negative result of the ST194-type *szM* PCR, a method which was previously suggested for the detection of highly virulent SEZ in swine [28].

## 4. Discussion and Conclusions

*S. equi* subsp. *zooepidemicus* is known to be primarily a pathogen of horses, dogs, and cats. It may reside in the tonsils of pigs, and although the prevalence of carriage is unknown, clinical disease is also considered rare. However, in Southeast Asian countries, particularly China, outbreaks of SEZ have caused severe epidemics since the 1970s, and zoonotic cases have been reported. Subsequently, the highly virulent strains causing the Asian epidemics have also emerged in North America and have caused serious losses in the pig sector. These significant cases were all caused by strains of a specific clonal lineage belonging to the ST194. The highly virulent ST194 variant has not yet been identified in Europe, and both strains of the two previously published European outbreak cases belonged to distinct, distantly related sequence types [20, 26]. For these reasons, we considered it important to investigate in more detail a small outbreak of sow abortion and sudden death in an organic farm in Western Hungary and by this contributing to the understanding of an emerging swine pathogen. To our knowledge, this is the first documentation and report of SEZ infection outbreak in swine in Central and Eastern Europe.

Genomic analysis of strains from the outbreak described here confirmed a non-ST194 type SEZ with some ubiquitous virulence markers compared to most SEZ isolates [6, 18]. Although the role of most markers or their certain combinations in the virulence of SEZ strains remains to be elucidated, it appears that the presence of putative virulence genes identified in highly virulent ST194 strains is not a prerequisite for the development of a severe clinical picture. This assumption is supported by the fact that the non-ST194 strains isolated by Chen and co-workers [14] from an outbreak in Indiana, USA, had a similarly modest virulence profile compared to the isolates described in this study. However, only disease models and infection experiments can confirm such presumptions. Also, comparative genomics studies are warranted to better understand genetic factors contributing to the higher virulence of SEZ ST194 and unrelated, yet still epizootic lineages in the pig host.

Clinical signs, pathological lesions, and microbiological findings during the recent outbreak described here were suggestive of septicaemia of bacterial origin, later determined to be caused by SEZ. The observed clinical signs and the course of the disease during the recent outbreak were grossly in line with those described in the highly virulent cases: rapid decline in the animal general condition, characterized by fever, loss of appetite, dyspnoea, ataxia and recumbency, and abortion in some cases, followed by death of the affected sows [10, 12, 15]. Such non-specific clinical picture could be associated with other relevant diseases, like classical and African swine fever, PRRS, and Aujeszky's disease, among others. The concomitant infection with these pathogens also could serve as predisposing factor for acute bacterial septicaemias. In our case, however, the subsequent testing for these pathogens helped to rule out all of them. Similarly, the testing of both sows and aborted foetuses excluded the presence of pathogens regularly involved in abortion. The on-site necropsy findings were non-specific, but still in accordance with those described both in highly virulent and unrelated American and European SEZ cases [14, 20, 26] and suggesting septicaemia. A more thorough pathological comparison was hurdled by the unfortunate lack of histopathological samples. During the routine laboratory examination of microbiological samples, no special culturing conditions were applied, and no targeted genetic analysis was carried out, so the presence of other fastidious bacteria could not be excluded.

The source of infection was not investigated in this outbreak, since SEZ could be presented as an opportunistic pathogen of the normal flora on the mucous membranes of swine. Introduction of SEZ by other carrier species is also possible, such as dogs, cats, and rodents, which may have occasionally come into contact with the pigs through their open open-air paddocks.

In any case, predisposing factors seem to play important role in the development and severity of SEZ infections. External stressors like heat stress, tissue damage, and transportation[2] or other predisposing events (e.g., water supply disturbances for even a short period of time) may help the pathogen to invade and combat the immune system. The outbreak occurred between 15th to 31st of August 2023, during which period the average daily temperature was over 20°C, and the daily maximum over 30°C for many consecutive days and initiated by arrival of a strong warm weather front. Such sudden weather change and subsequent persistent heat stress is suggested as a key predisposing factor for this outbreak since no other stressors could be identified. A similar conclusion was proposed in the case of the German outbreak, where the ventilation might have failed to guarantee appropriate air exchange rates under hot summer conditions [20].

The immediate isolation of diseased animals from healthy ones is essential in the case of SEZ outbreaks, because clinical trials suggested that direct contact is required for pathogen transmission [15]. Such measures might have helped the producer to prevent the spreading of the pathogen, reflected in the low incidence of the diseased animals. The other key factor might have been the quick application of an appropriate antibiotic therapy, in the form of amoxicillin trihydrate, which choice was later confirmed by the susceptibility test results. Beta-lactam antibiotics proved to be effective in the treatment of SEZ in previous swine cases too [12, 13, 20]. Besides reducing mortality rate, a well-chosen and timely course of antibiotics helps the herd to seroconvert, and treated animals no longer pose a threat to naïve, healthy ones [38]. The applied amoxicillin therapy brought an abrupt end to the outbreak, and no further disease was observed since then.

## Figures and Tables

**Table 1:** Summary of findings from ancillary tests following post-mortem sampling.

Strain	Isolation site	Catalase test	Haemolysis	API 20 Strep	SEZ-SEE mPCR	szM PCR	AST	WGS
S23-21851	Foetal lung	Negative	Beta	SEZ	SEZ	Negative	No	No
S23-21852	Foetal lung	Negative	Beta	SEZ	SEZ	Negative	Yes	Yes
S23-21853	Sow, liver	Negative	Beta	SEZ	SEZ	Negative	No	No
S23-21854	Sow, spleen	Negative	Beta	SEZ	SEZ	Negative	Yes	Yes

SEZ, *Streptococcus. equi* subsp. *zooepidemicus*; SEE, *Streptococcus. equi* subsp. *equi*; mPCR, multiplex polymerase chain reaction; szM PCR, *Streptococcus. equi* subsp. *zooepidemicus* M protein gene PCR; AST, antimicrobial susceptibility testing; WGS, whole-genome sequencing.

**Table 2:** Antimicrobial susceptibility testing results of the investigated *S. equi* subsp. *zooepidemicus* isolates.

Antimicrobial compound	Range of observation (mg/L)	S23-21852		S23-21854	
		(mg/L)	Clinical category	(mg/L)	Clinical category
Ampicillin	0.125–8	≤0.125	S	≤ 0.125	S
Cefquinome	2–4	≤2	S	≤ 2	S
Ceftiofur	0.125–4	≤0.125	S	≤ 0.125	S
Cefazolin	1–8	≤1	S	≤ 1	S
Clindamycin	0.125–2	≤0.125	S	= 0.25	S
Cefoxitin	2–4	≤2	S	≤ 2	S
Doxycycline	0.125–8	≤0.125	S	= 0.25	S
Enrofloxacin	0.0625–2	= 2	R	= 0.5	I
Erythromycin	0.125–4	= 0.25	S	≤ 0.125	S
Florfenicol	1–8	≤1	S	≤ 1	S
Gentamicin	1–8	= 4	S	= 2	S
Gentamicin (high level)	500	≤500	S	≤ 500	S
Neomycin	8–16	= 16	I	= 16	I
Nitrofurantoin	32–64	≤32	S	≤ 32	S
Oxacillin	0.125–4	≤0.125	S	≤ 0.125	S
Penicillin G	0.125–8	≤0.125	S	≤ 0.125	S
Rifampicin	0.0625–4	= 0.25	S	= 0.25	S
Trimethoprim/sulfmethoxazole	0.25/4.75−4/76	= 1/19	S	= 1/19	S
Tetracycline	0.25–8	= 4	I	= 4	I
Vancomycin	0.5–16	≤0.5	S	≤ 0.5	S

S, susceptible; I, intermediate resistance; R, resistant.

## Data Availability

The data that support the findings of this study are available in the Supplementary Material file of this article. Sequencing data of *S. equi* subsp. *zooepidemicus* strains obtained in this study have been submitted to the NCBI Sequence Read Archive database under the BioProject accession number PRJNA1086057.

## Supplementary Materials

Supplementary Materials Table S1: accession and reference of the 14 selected virulence determinant genes investigated in *Streptococcus equi* subsp. *zooepidemicus* and subsp. equi isolates. Table S2: presence of 14 selected virulence determinant genes in the investigated *Streptococcus equi* subsp. *zooepidemicus* and subsp. equi isolates.
