# Supplementary material for: Abortion and Lethal Septicaemia in Sows Caused by a Non-ST194 Streptococcus equi subsp. zooepidemicus
Source: Transbound Emerg Dis. 2024 Jul 23;2024:4008946. doi: 10.1155/2024/4008946 (PMC12016921; doi:10.1155/2024/4008946)
Supplement: Supplementary Materials — Table S1: accession and reference of the 14 selected virulence determinant genes investigated in Streptococcus equi subsp. zooepidemicus and subsp. equi isolates. Table S2: presence of 14 selected virulence determinant genes in the investigated Streptococcus equi subsp. zooepidemicus and subsp. equi isolates. [file 4008946.f1.docx]

**Supplementary Material** to Albert et al., (2024) - Abortion and Lethal Septicaemia in Sows Caused by a Non-ST194 *Streptococcus equi* subsp. *zooepidemicus* – a case report

**Supplemental Table S1.** Accession and reference of the 14 selected virulence determinant genes investigated in *Streptococcus equi* subsp. *zooepidemicus* and subsp. *equi* isolates.

| **Name** | **Abbrev.** | **Accession** | **Reference** | **DOI** |
| --- | --- | --- | --- | --- |
| Streptococcus M-like protein gene *szP* | *szP* | CP002904 | Ma et al., 2013. | 10.1186/1471-2164-14-377 |
| Streptococcus M-like protein gene *szM* | *szM* | CP002904 | Ma et al., 2013. | 10.1186/1471-2164-14-377 |
| Streptococcus M-like protein gene *seM* | *seM* | NC_12471 | Holden et al., 2009. | 10.1371/journal.ppat.1000346 |
| Streptococcal protective antigen *spaZ* | *spaZ* | CP002904 | Ma et al., 2013. | 10.1186/1471-2164-14-377 |
| Fic domain-containing protein gene *bifA* | *bifA* | CP002904 | Ma et al., 2013. | 10.1186/1471-2164-14-377 |
| Fimbrial subunit protein-coding gene *fszF* | *fszF* | CP002904 | Ma et al., 2013. | 10.1186/1471-2164-14-377 |
| Streptodornase type D gene | *sdzD* | CP002904 | Ma et al., 2013. | 10.1186/1471-2164-14-377 |
| Strep. equi ssp. equi superantigen coding gene H | *seeH* | NC_12471 | Holden et al., 2009. | 10.1371/journal.ppat.1000346 |
| Strep. equi ssp. equi superantigen coding gene I | *seeI* | NC_12471 | Holden et al., 2009. | 10.1371/journal.ppat.1000346 |
| Strep. equi ssp. equi superantigen coding gene L | *seeL* | NC_12471 | Holden et al., 2009. | 10.1371/journal.ppat.1000346 |
| Strep. equi ssp. equi superantigen coding gene M | *seeM* | NC_12471 | Holden et al., 2009. | 10.1371/journal.ppat.1000346 |
| Streptococcal superantigen coding gene F | *szeF* | CABY00000000 | Paillot et al., 2010. | 10.1128/IAI.00751-10 |
| Streptococcal superantigen coding gene N | *szeN* | CABY00000000 | Paillot et al., 2010. | 10.1128/IAI.00751-10 |
| Streptococcal superantigen coding gene P | *szeP* | CABY00000000 | Paillot et al., 2010. | 10.1128/IAI.00751-10 |

**Supplemental Table S2.** Presence of 14 selected virulence determinant genes in the investigated *Streptococcus equi* subsp. *zooepidemicus* and subsp. *equi* isolates. Strain IDs are as seen in Figure 1. MLST: multilocus sequence type; ID: sequence identity; QC: query coverage. (*) The ST194-specfic variant of the M-protein gene.

|  |  |  | ***szP*** | | ***szM**** | | ***seM*** | | ***spaZ*** | | ***bifA*** | | ***fszF*** | | ***sdzD*** | | ***seeH*** | | ***seeI*** | | ***seeL*** | | ***seeM*** | | ***szeF*** | | ***szeN*** | | ***szeP*** | |
| --- | --- | --- | --- | --- | --- | --- | --- | --- | --- | --- | --- | --- | --- | --- | --- | --- | --- | --- | --- | --- | --- | --- | --- | --- | --- | --- | --- | --- | --- | --- |
| **Strain ID as seen in Figure 1** | **MLST** | **GenBank Accessions** | **ID (%)** | **QC (%)** | **ID (%)** | **QC (%)** | **ID (%)** | **QC (%)** | **ID (%)** | **QC (%)** | **ID (%)** | **QC (%)** | **ID (%)** | **QC (%)** | **ID (%)** | **QC (%)** | **ID (%)** | **QC (%)** | **ID (%)** | **QC (%)** | **ID (%)** | **QC (%)** | **ID (%)** | **QC (%)** | **ID (%)** | **QC (%)** | **ID (%)** | **QC (%)** | **ID (%)** | **QC (%)** |
| SEZ/TN-714097/USA, Tennessee/2019/pig | ST194 | CP046042 | 100 | 100 | 100 | 100 | - | - | 100 | 100 | 100 | 100 | 100 | 100 | 100 | 100 | - | - | - | - | - | - | - | - | - | - | - | - | - | - |
| SEZ/NVSLTN-LUNG2/USA, Tennessee/2019/pig | ST194 | WOGB00000000 | 100 | 100 | 100 | 100 | - | - | 100 | 100 | 100 | 100 | 100 | 100 | 100 | 100 | - | - | - | - | - | - | - | - | - | - | - | - | - | - |
| SEZ/NVSLTN-LUNG3/USA, Tennessee/2019/pig | ST194 | WOGC00000000 | 100 | 100 | 100 | 100 | - | - | 100 | 100 | 100 | 100 | 100 | 100 | 100 | 100 | - | - | - | - | - | - | - | - | - | - | - | - | - | - |
| SEZ/166440/Canada/2019/pig | ST194 | n/a | 100 | 100 | 100 | 100 | - | - | 100 | 100 | 100 | 100 | 100 | 50.1 | 100 | 100 | - | - | - | - | - | - | - | - | - | - | - | - | - | - |
| SEZ/166438/Canada/2019/pig | ST194 | n/a | 100 | 100 | 100 | 100 | - | - | 100 | 100 | 100 | 90.7 | 100 | 100 | 100 | 99.8 | - | - | - | - | - | - | - | - | - | - | - | - | - | - |
| SEZ/NVSLTN-TB1/USA, Tennessee/2019/pig | ST194 | WOGD00000000 | 100 | 100 | 100 | 100 | - | - | 100 | 100 | 100 | 100 | 100 | 100 | 100 | 100 | - | - | - | - | - | - | - | - | - | - | - | - | - | - |
| SEZ/A02199501/USA, Pennsylvania/NA/pig | ST194 | JABDID000000000 | 100 | 100 | 100 | 100 | - | - | 100 | 100 | 100 | 100 | 100 | 100 | 100 | 100 | - | - | - | - | - | - | - | - | - | - | - | - | - | - |
| SEZ/CY/China/1998/pig | ST194 | CP006770.1 | 100 | 100 | 100 | 100 | - | - | 100.0 | 100.0 | 100 | 100 | 100 | 100 | 100 | 100 | - | - | - | - | - | - | - | - | - | - | - | - | - | - |
| SEZ/ATCC 35246/China/1975/pig | ST194 | CP002904.1 | 100 | 100 | 100 | 100 | - | - | 100 | 100 | 100 | 100 | 100 | 100 | 100 | 100 | - | - | - | - | - | - | - | - | - | - | - | - | - | - |
| SEZ/20854/Thailand/2005/human | ST194 | JAGSOD000000000 | 100 | 100 | 100 | 100 | - | - | 100 | 100 | 100 | 100 | 100 | 100 | 100 | 100 | - | - | - | - | - | - | - | - | - | - | - | - | - | - |
| SEZ/58622/Thailand/2012/human | ST194 | JAGSOF000000000 | 100 | 100 | 100 | 100 | - | - | 100 | 100 | 100 | 100 | 100 | 100 | 100 | 100 | - | - | - | - | - | - | - | - | - | - | - | - | - | - |
| SEZ/35935/Thailand/2011/human | ST194 | JAGSOE000000000 | 100 | 100 | 100 | 100 | - | - | 100 | 100 | 100 | 100 | 100 | 100 | 100 | 100 | - | - | - | - | - | - | - | - | - | - | - | - | - | - |
| SEZ/IA-61192/USA, Iowa/2019/horse | ST329 | WOFY00000000 | 96.0 | 59.3 | 99.0 | 25.2 | - | - | 98.0 | 100.0 | 100 | 100 | 100 | 100 | 100 | 100 | - | - | - | - | - | - | - | - | - | - | - | - | - | - |
| SEZ/NDL/The Netherlands/2019/pig | ST326 | n/a | 94.0 | 60.1 | 100 | 100 | - | - | 91.0 | 59.1 | - | - | - | - | - | - | - | - | - | - | - | - | - | - | - | - | - | - | - | - |
| SEZ/Sz105/Denmark/2007/horse | ST140 | JATZ00000000 | 94.0 | 59.3 | 96.0 | 56.9 | - | - | 94.0 | 62.9 | - | - | - | - | 100 | 100 | - | - | - | - | - | - | - | - | - | - | - | - | - | - |
| SEZ/IN-6992/USA, Indiana/2021/pig | ST132 | CP073275 | 96.0 | 60.1 | - | - | - | - | 92.0 | 60.5 | - | - | 99.0 | 62.3 | 100.0 | 100.0 | - | - | - | - | - | - | - | - | - | - | - | - | - | - |
| SEZ/SEZ_18-036/USA, Texas/2018/horse | ST200 | CP078012 | 90.0 | 100.0 | - | - | - | - | 93.0 | 70.1 | - | - | - | - | 100 | 100 | - | - | - | - | - | - | - | - | 99.0 | 96.3 | - | - | - | - |
| SEZ/BHS5/United Kingdom/2001/dog | ST123 | CABY00000000 | 92.0 | 100 | - | - | - | - | 93.0 | 51.4 | - | - | - | - | - | - | - | - | - | - | - | - | - | - | 100 | 100 | 100 | 100 | 100 | 100 |
| SEZ/Sz57/Denmark/2007/horse | ST96 | JAUA00000000 | 93.0 | 100.0 | 98.0 | 27.0 | - | - | 94.0 | 60.5 | - | - | - | - | 100 | 100 | - | - | - | - | - | - | - | - | 96.0 | 100 | - | - | - | - |
| SEZ/Sz35/Denmark/2007/horse | ST203 | JAUB00000000 | 89.0 | 100.0 | - | - | - | - | 93.0 | 51.4 | 100 | 100 | 100 | 100 | - | - | - | - | - | - | - | - | - | - | 96.0 | 100 | - | - | - | - |
| SEZ/AZ-45470/USA, Arizona/2019/pig | ST340 | CP046041 | 96.0 | 60.1 | - | - | - | - | 93.0 | 51.4 | - | - | - | - | - | - | - | - | - | - | - | - | - | - | - | - | - | - | - | - |
| SEZ/S23-21852/Hungary/2023/pig | ST138 | n/a | 95.0 | 100.0 | 94.0 | 75.6 | - | - | 92.0 | 59.1 | - | - | - | - | 100 | 100 | - | - | - | - | - | - | - | - | - | - | - | - | - | - |
| SEZ/S23-21854/Hungary/2023/pig | ST138 | n/a | 95.0 | 100.0 | 94.0 | 75.6 | - | - | 92.0 | 59.1 | - | - | - | - | 100 | 100 | - | - | - | - | - | - | - | - | - | - | - | - | - | - |
| SEZ/NCTC4676/United Kingdom/1900-1936/cow | ST214 | UHFI00000000 | 86.0 | 100.0 | - | - | - | - | 100 | 100 | - | - | - | - | 100 | 100 | - | - | - | - | - | - | - | - | - | - | - | - | - | - |
| SEZ/ISU54485/USA, Illinois/2017/cat | ST40 | WMAB00000000 | 93.0 | 38.9 | 99.0 | 25.2 | - | - | 92.0 | 59.1 | - | - | - | - | 100 | 100 | - | - | - | - | - | - | - | - | - | - | - | - | - | - |
| SEZ/SEZ_19-045/USA, Texas/2013/horse | ST15 | JAHLGY000000000 | 96.0 | 59.2 | 98.0 | 25.6 | - | - | 91.0 | 51.4 | - | - | - | - | 100 | 100 | - | - | - | - | - | - | - | - | - | - | - | - | - | - |
| SEZ/SzAM35/USA, Kentucky/2007/horse | ST65 | JATY00000000 | 94.0 | 59.3 | - | - | - | - | 92.0 | 60.5 | - | - | 99.0 | 100 | - | - | - | - | - | - | - | - | - | - | - | - | - | - | - | - |
| SEZ/NCTC11854/United Kingdom/1983/human | ST65 | LR590471 | 94.0 | 59.3 | - | - | - | - | 92.0 | 60.5 | - | - | 99.0 | 100.0 | - | - | - | - | - | - | - | - | - | - | - | - | - | - | - | - |

**Supplemental Table S2.** (continued) Presence of 14 selected virulence determinant genes in the investigated *Streptococcus equi* subsp. *zooepidemicus* and subsp. *equi* isolates. Strain IDs are as seen in Figure 1. MLST: multilocus sequence type; ID: sequence identity; QC: query coverage. (*) The ST194-specfic variant of the M-protein gene.

|  |  |  | ***szP*** | | ***szM**** | | ***seM*** | | ***spaZ*** | | ***bifA*** | | ***fszF*** | | ***sdzD*** | | ***seeH*** | | ***seeI*** | | ***seeL*** | | ***seeM*** | | ***szeF*** | | ***szeN*** | | ***szeP*** | |
| --- | --- | --- | --- | --- | --- | --- | --- | --- | --- | --- | --- | --- | --- | --- | --- | --- | --- | --- | --- | --- | --- | --- | --- | --- | --- | --- | --- | --- | --- | --- |
| **Strain ID as seen in Figure 1** | **MLST** | **GenBank Accessions** | **ID (%)** | **QC (%)** | **ID (%)** | **QC (%)** | **ID (%)** | **QC (%)** | **ID (%)** | **QC (%)** | **ID (%)** | **QC (%)** | **ID (%)** | **QC (%)** | **ID (%)** | **QC (%)** | **ID (%)** | **QC (%)** | **ID (%)** | **QC (%)** | **ID (%)** | **QC (%)** | **ID (%)** | **QC (%)** | **ID (%)** | **QC (%)** | **ID (%)** | **QC (%)** | **ID (%)** | **QC (%)** |
| SEZ/ISU54026/USA, Iowa/2019/horse | ST71 | WMAC00000000 | 93.0 | 59.2 | 90.0 | 100 | - | - | 94.0 | 59.1 | - | - | - | - | 100 | 100 | - | - | - | - | - | - | - | - | - | - | - | - | - | - |
| SEZ/NCTC4675/United Kingdom/1935/cow | ST39 | LR594033 | 95.0 | 59.3 | 95.0 | 54.0 | - | - | 93.0 | 51.4 | - | - | - | - | - | - | - | - | - | - | - | - | - | - | - | - | - | - | - | - |
| SEZ/Sz5/Denmark/2007/horse | ST303 | JAUC00000000 | 90.0 | 60.1 | - | - | - | - | 93.0 | 51.4 | - | - | - | - | - | - | - | - | - | - | 99.0 | 100 | 100 | 100 | - | - | - | - | - | - |
| SEE/19/Ireland/1985/horse | ST179 | LMTY00000000 | 95.0 | 59.3 | - | - | 100.0 | 100.0 | 94.0 | 59.1 | - | - | - | - | 99.0 | 100 | 100 | 100 | 100 | 100 | 100 | 100 | 100 | 100 | - | - | - | - | - | - |
| SEE/EQUI0203/Netherlands/1990/NA | ST395 | CWEQ00000000 | 95.0 | 59.3 | - | - | 100.0 | 100.0 | 94.0 | 59.1 | - | - | - | - | 99.0 | 100 | 100 | 100 | 100 | 99.9 | 100 | 100 | 100 | 100 | - | - | - | - | - | - |
| SEE/EQUI0085/United Kingdom/2007/horse | ST179 | CWAI00000000 | 97.0 | 59.3 | - | - | 100.0 | 100.0 | 94.0 | 59.1 | - | - | - | - | 99.0 | 100 | 100 | 100 | 100 | 99.9 | 100 | 100 | 100 | 100 | - | - | - | - | - | - |
| SEE/EQUI0024/United Kingdom/2006/horse | ST151 | CWFX00000000 | 95.0 | 59.3 | - | - | 100.0 | 100.0 | 94.0 | 59.1 | - | - | - | - | 99.0 | 100 | 100 | 100 | 100 | 99.9 | 100 | 100 | 100 | 100 | - | - | - | - | - | - |
| SEE/EQUI0139/United Kingdom/2010/horse | ST151 | CWCB00000000 | 95.0 | 59.3 | - | - | 100.0 | 100.0 | 94.0 | 59.1 | - | - | - | - | 99.0 | 100 | 100 | 100 | 100 | 99.9 | 100 | 100 | 100 | 100 | - | - | - | - | - | - |
| SEE/EQUI0126/United Kingdom/2008/horse | ST151 | CWBP00000000 | 95.0 | 59.3 | - | - | 100.0 | 100.0 | 94.0 | 59.1 | - | - | - | - | 99.0 | 100 | 100 | 100 | 100 | 99.9 | 100 | 100 | 100 | 100 | - | - | - | - | - | - |
| SEE/470_002/Sweden/2015/horse | ST179 | JAFKDV000000000 | 95.0 | 59.3 | - | - | 100.0 | 100.0 | 94.0 | 59.1 | - | - | - | - | 99.0 | 100 | 100 | 100 | 100 | 99.9 | 100 | 100 | 100 | 100 | - | - | - | - | - | - |
| SEE/EQUI0206/Belgium/2010/horse | ST179 | CWEX00000000 | 95.0 | 59.3 | - | - | 99.0 | 100.0 | 94.0 | 59.1 | - | - | - | - | 99.0 | 100 | 100 | 100 | 100 | 99.9 | 100 | 100 | 100 | 100 | - | - | - | - | - | - |
